# Atypical Meningioma of the Sinonasal Tract

**DOI:** 10.7759/cureus.14908

**Published:** 2021-05-08

**Authors:** Linnea C Fechtner, Philip R Persino, Mark S Burke

**Affiliations:** 1 Otolaryngology, University at Buffalo, Buffalo, USA; 2 Otolaryngology - Head and Neck Surgery, University at Buffalo, Buffalo, USA; 3 Head and Neck-Plastics and Reconstructive Surgery, Erie County Medical Center, Buffalo, USA

**Keywords:** olfactory bulb, atypical meningioma, sinonasal meningioma, ethic, radiation

## Abstract

This case report describes a patient with intellectual disability who presented with a neglected midline nasal mass eroding the anterior skull base, which was found to be a Grade II meningioma likely of the olfactory bulb. Points of interest include differential diagnosis of this atypical mass of the sinonasal tract, as well as decision-making in balancing appropriate management and quality of life in a patient with developmental delay who could not make decisions for herself. Literature review regarding the role of adjuvant radiation based on final diagnosis and extent of disease suggests that radiation can improve locoregional control and overall survival with atypical meningioma. Lack of clear information in the literature on these rare conditions can lead to poor understanding on the part of the treatment team and the healthcare proxies who are making decisions, making goals of care discussions and medical decision-making challenging. This case report seeks to add to the available data on management of atypical meningiomas of the sinonasal tract and olfactory bulb.

## Introduction

Meningiomas are the most commonly reported primary intracranial neoplasms in adults, comprising over one-third of all central nervous system tumors [1]. The World Health Organization (WHO) 2007 classification criteria divide meningiomas into Grade I (benign), Grade II (atypical), and Grade III (malignant); with these new criteria, about 20%-35% of meningiomas have now been classified as atypical [2]. There is limited data on clinical behavior, outcomes, and optimal management of atypical meningiomas. While the majority of meningiomas fall within the realm of neurosurgical resection, extracranial meningiomas can rarely present in the sinonasal tract and are first evaluated by otolaryngologists; these include primary sinonasal meningiomas and olfactory groove meningiomas with extension to the paranasal sinuses. Despite immunohistochemical staining, the differential diagnosis of a midline nasal mass involving the skull base remains broad. The prevalence of meningiomas presenting in the head and neck with atypical meningioma on histopathology is unknown, given independent rarity of those two events. This case report describes an unusual presentation of atypical olfactory groove meningioma in a patient with baseline intellectual disability, as well as a literature review regarding the role of adjuvant radiation based on final diagnosis and extent of disease.

## Case presentation

The patient was a 64-year-old female with a history of severe intellectual disability and seizures who presented with a midline nasal mass that had been steadily increasing in size for the prior four months. On physical exam, the mass was visible and palpable deep to the skin along the left nasal dorsum, leading to left orbital proptosis and severe telecanthus. At baseline, the patient was able to make her needs known with limited vocabulary, was cooperative with her own care, and lived in a nursing facility.

**Figure 1:**
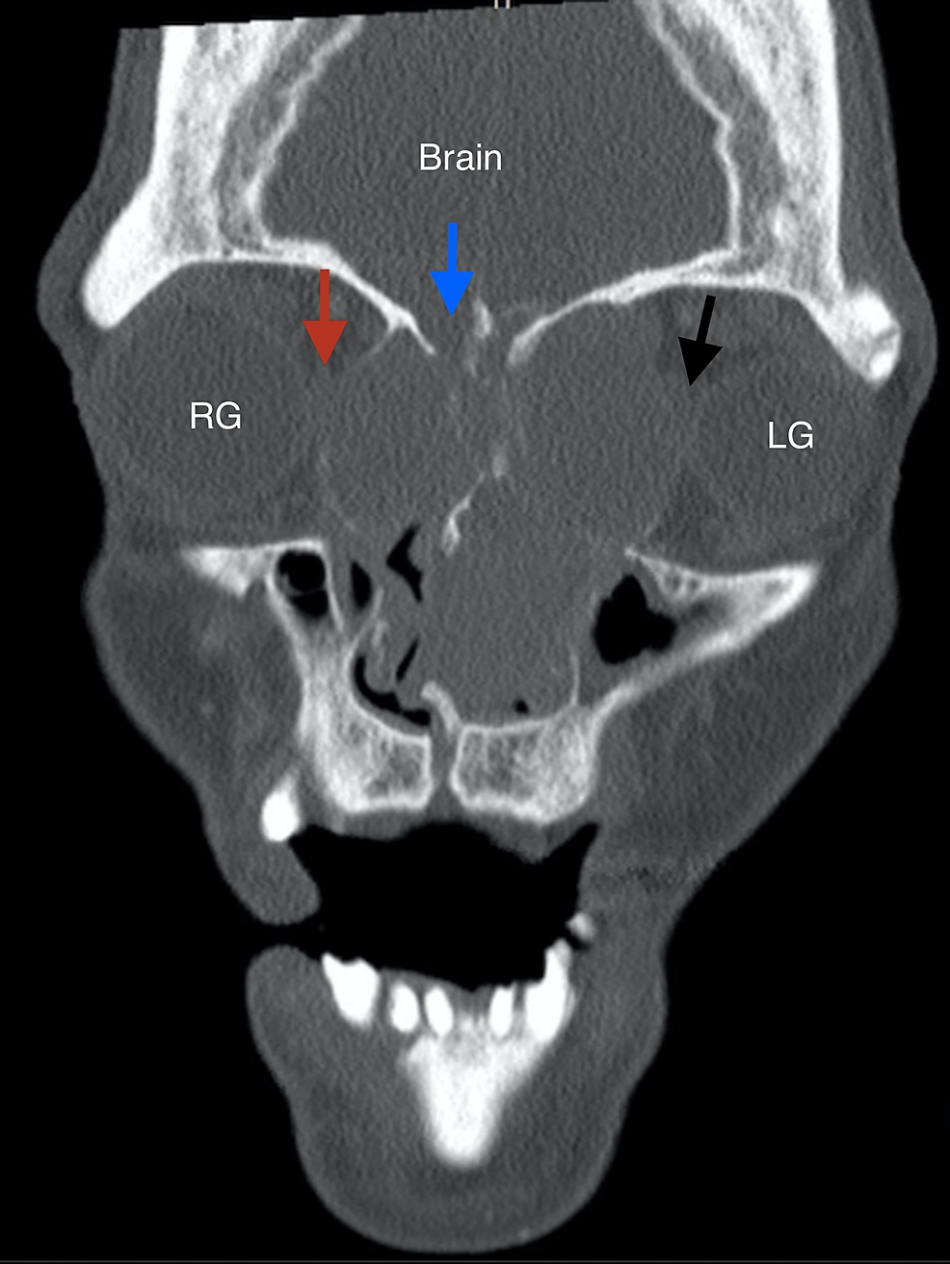
CT bone window, coronal view, at the level of the orbit, showing proptosis of bilateral globes. The red arrow indicates the invasion of the right orbital cavity, which had lateralized the right medial orbital wall, causing proptosis of the right globe (RG). The black arrow indicates similar invasion of the left orbit, with erosion of the left medial orbital wall and proptosis of the left globe (LG). The blue arrow indicates erosion of the anterior skull base, lateral to the cribriform plate.

CT scan with intravenous (IV) contrast showed 5.8 x 5.4 x 4.8cm homogenously enhancing lobulated mass centered in the midline anterior cranial fossa near the olfactory groove, frontal sinus, and anterior ethmoid sinus, demonstrating marked scalloping and thinning of the bone, with 1.3 x 1.3cm extension into the intracranial space along the anterior cranial fossa (Figure 1). The mass was noted to be causing bilateral globe proptosis, and extending into bilateral middle meatus and left anterior nasal cavity. 

**Figure 2:**
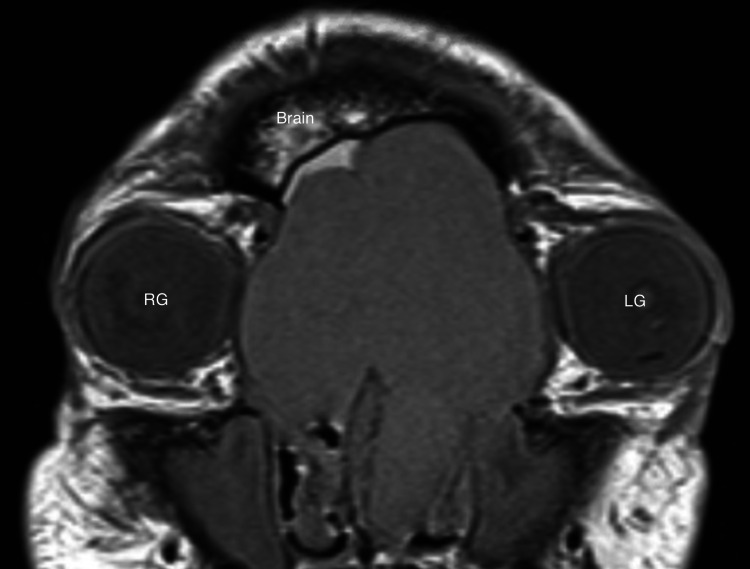
MRI on presentation, coronal view, T1 MRI with gadolinium demonstrating mass effect on skull base and globes. RG: right globe: LG: left globe.

**Figure 3:**
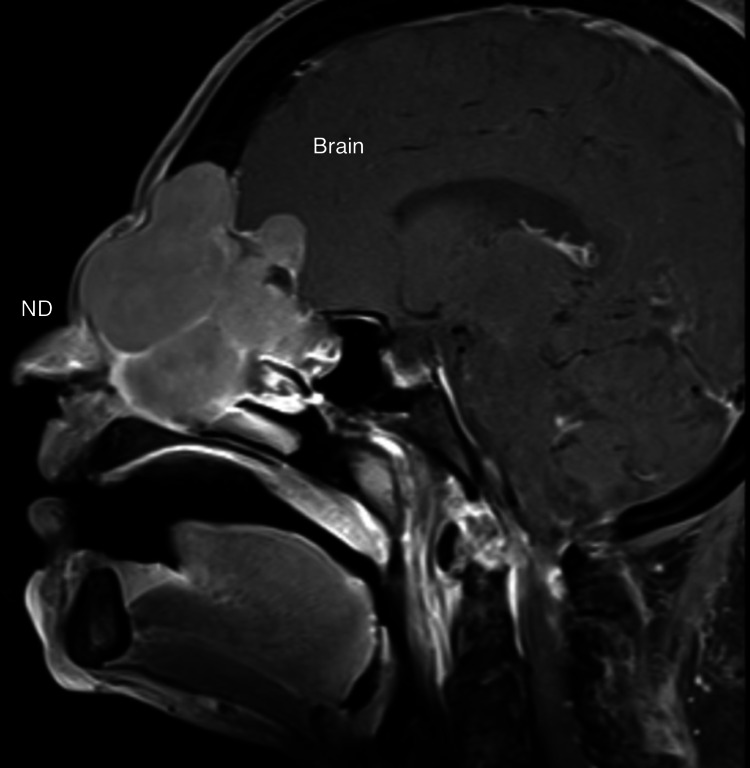
MRI on presentation, sagittal, T1 MRI with gadolinium, demonstrating mass effect on frontal lobe. Nasal dorsum (ND) is labeled. Enhancing mass does not erode though nasal dorsum or into oral cavity.

Biopsy results demonstrated a low-grade neoplasm of uncertain lineage, which could not exclude meningioma, synovial cell sarcoma, and mesenchymal chondrosarcoma. Further staining results showed epithelial membrane antigen (EMA) was weakly positive, S100 was diffusely positive, CK7 was positive, and CD99 was positive. Follow-up magnetic resonance imaging (MRI) confirmed the CT findings, and showed that the lesion was hypointense on T1, mildly hyperintense on T2, and had displaced homogeneous avid enhancement (Figures 2, 3).

The proposed differential at that time was meningioma, esthesioneuroblastoma, lymphoma, or sinonasal carcinoma. The goals of care discussion with the patient’s family and healthcare proxy was challenging in this case, as it was unclear how to frame the risks and benefits of an aggressive resection and reconstruction in a non-verbal, minimally interactive patient with a mass of uncertain prognosis. It was difficult to determine the best thing for this particular patient’s quality of life. The concern with leaving the mass untreated was for continued growth of the lesion, worsening proptosis and mass effect on the brain and brain stem, leading to death. Given the unknown pathology of the lesion, it was difficult to prognosticate on the timeline of these events, or on the response of this mass to non-surgical management, such as radiation and chemotherapy. The risks of removal included postoperative leak of cerebrospinal fluid (CSF) and meningitis. Given the erosion of the bone of the skull base seen on CT, excision of the mass would certainly require intraoperative repair of the skull base, which can still lead to persistent CSF leak in 1.6% of cases and meningitis in 1.1% of cases, based on the current literature in endoscopic resection of skull base lesions [3]. Surgical resection with possible post-operative radiation and chemotherapy was offered despite the lack of definitive pathology, given the progressive nature of her disease. More aggressive reconstruction with microvascular free flap and cartilage grafts was offered, which would allow for more reliable repair of the skull base and for reconstruction of the likely cosmetic defect after resection of the nasal bones, but this was declined by the family. Decision-making was performed in conjunction with her nieces, who were her healthcare proxies.

The patient underwent resection of the mass via modified Weber-Ferguson incision as well as bicoronal flap, bifrontal craniotomy with excision of tumor and anterior cranial fossa base with repair with pericranium and fascia lata grafts in a combined approach by Neurosurgery and Head and Neck Surgery. The tumor was noted to be closely associated with the cranial part of the nasal septum and the medial left orbit, but was not grossly invading these structures. The majority of the destruction of the skull base appeared to be lateral to the cribriform plate on the left. The resected area included left ethmoid air cells, left middle turbinate, right middle and superior turbinate, superior nasal septum, nasal bones, and bilateral medial orbital walls. The anterior skull base defect was repaired with an anteriorly-based pericranial graft and fascia lata grafts harvested from the thigh. Because there was no resection of the overlying skin, the fascial incision was closed primarily with minimal cosmetic defect, other than saddle nose deformity of the nose due to resection of the nasal bones and superior nasal septum. No masses or abnormal enhancement on postoperative CT performed during her hospital say suggested current or residual disease.

The final pathology result showed atypical meningioma with positive margins, including bony invasion of the posterior nasal septal margin and cribriform plate as well as submucosal tissue of the anterior septal margin. Immunostaining was positive for S100, EMA, and CD56.

**Figure 4:**
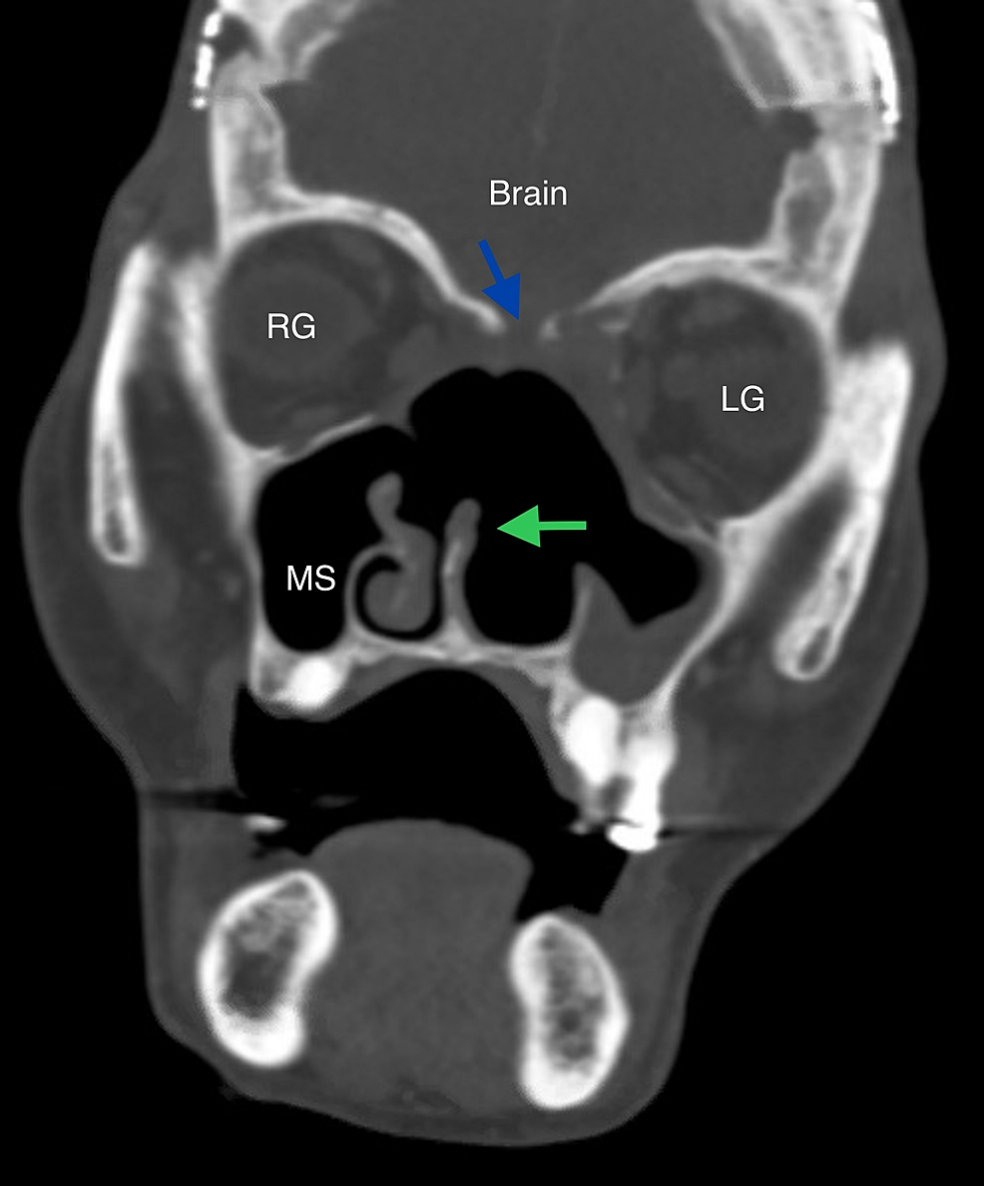
CT scan, bone window, coronal view, at the level of the orbits, one year after resection. Blue arrow once again indicates bony skull base defect lateral to the cribriform plate. Right globe (RG) and left globe (LG) no longer demonstrate proptosis. Green arrow indicates remnant of nasal septum. Right maxillary sinus (MS) and abutting right inferior turbinate are the only remaining landmarks in the nasal cavity.

At the initial postoperative visit one week after discharge from the hospital, further postoperative management was discussed. Because of diagnosis of atypical meningioma and invasion of bone on final pathology, radiation therapy was recommended; however, given concern over side effects of radiation and desire to limit aggressive care at that time, decision was made by the patient’s healthcare proxies to forgo radiation. There was no residual or recurrent disease noted at the one-year follow-up visit. Postoperative CT findings at one year showed no residual disease or recurrence (Figure 4).

At the two-year follow-up visit, the patient was noted to have a recurrence with worsening proptosis. Options of further resection and reconstruction with anterolateral thigh free flap, as well as palliative care and observation were discussed. The patient’s healthcare proxies chose to proceed with surgery. Postoperative course was complicated by poor wound healing and flap breakdown, which was managed conservatively without return to the operating room. The patient subsequently expired within 14 days after discharge back to the nursing home.

## Discussion

Recurrent rates for atypical and anaplastic meningioma range between 9% and 50% after gross total resection (GTR) and between 36%-83% after subtotal resection (STR) [4]. Bone involvement was associated with an increased rate of disease progression (p = 0.001) and decreased survival (p = 0.04). 78% of patients with bone involvement at primary diagnosis had tumor recurrence within bone, whereas only 25% of patients without evidence of bone invasion at primary diagnosis experienced osseous recurrence [5].

One meta-analysis showed median five-year progression-free survival (PFS) and overall survival (OS) after adjuvant radiotherapy were 54.2% and 67.5% for atypical meningioma. Most studies failed to demonstrate a statistically significant prognostic benefit for adjuvant radiotherapy in atypical meningioma, but local control was improved, especially after subtotal resection [6]. An additional study of 162 adults with high-grade meningioma and 99 with atypical meningioma showed survival benefit for adjuvant radiation in subgroup analysis of patients with high grade who underwent subtotal resection. However, for recurrent atypical and anaplastic meningioma, radiotherapy offered no improvement in PFS and OS [7].

Based on this data, radiation may have benefited progression-free survival and overall survival in this patient based on bony invasion and pathology of atypical meningioma. 

## Conclusions

Meningiomas can present rarely in the sinonasal tract. The majority of meningiomas are benign; however, atypical and anaplastic meningiomas warrant more aggressive treatment both intraoperatively and postoperatively. Progression-free survival and overall survival are improved with adjuvant radiation to prevent recurrence, particularly in those patients with involvement of bone shown on final pathology. Studies have not shown improvement in PFS and OS after radiation for recurrent disease. Post-operative radiation was not used in the case of this patient, as the patient’s family wanted to forego aggressive treatment in the immediate postoperative period. However, the patient’s healthcare proxies then chose to pursue surgery two years later for treatment of the recurrence, which indicates they were willing to pursue aggressive measures when indicated. Refusing postoperative radiation may have been due to a lack of understanding of its risks and benefits on the part of both the treatment team and the family. It can be difficult to explain the risks and benefits of radiation for a lesion that is pathologically benign but locally aggressive, such as in this case of an atypical meningioma. The rarity of this condition, particularly its presentation in the sinonasal tract, led to difficulty with obtaining and conveying the appropriate information to use to counsel the patient's family. This case report seeks to summarize the available information in the literature to help provide evidence to assist with future patient discussions. 
